# *Lactiplantibacillus plantarum* ZDY2013 Inhibits the Development of Non-Alcoholic Fatty Liver Disease by Regulating the Intestinal Microbiota and Modulating the PI3K/Akt Pathway

**DOI:** 10.3390/nu16070958

**Published:** 2024-03-27

**Authors:** Qiang Teng, Huihui Lv, Lingling Peng, Zhongyue Ren, Jiahui Chen, Lixue Ma, Hua Wei, Cuixiang Wan

**Affiliations:** 1 State Key Laboratory of Food Science and Resources, Nanchang University, 235 Nanjing East Road, Nanchang 330047, China; 2 Jiangxi-OAI Joint Research Institute, Nanchang University, 235 Nanjing East Road, Nanchang 330047, China

**Keywords:** NAFLD, *L. plantarum*, gut microbiota, PI3K/Akt pathway, LPS/NF-κB pathway

## Abstract

Non-alcoholic fatty liver disease (NAFLD) is a common chronic hepatic condition whose impact on human health is increasingly significant. The imbalance of the gut microbiome, linked to insulin resistance, heightened intestinal permeability, and pro-inflammatory reactions, may be the linchpin in the development of NAFLD. In our research, the impact of *Lactiplantibacillus plantarum* ZDY2013 administration for 12 weeks on gut microbiota dysbiosis induced by a high-fat, high-fructose, high-cholesterol (FHHC) diet in male C57BL/6n mice was investigated. Research results presented that the intervention of *L. plantarum* ZDY2013 in mice fed with the FHHC diet could restore their liver function and regulate oxidative stress. Compared to mice in the model group, the intervention of *L. plantarum* ZDY2013 significantly regulated the gut microbiota, inhibited the LPS/NF-κB pathway, and led to a lower level of colonic inflammation in the mice administered with *L. plantarum* ZDY2013. It also improved insulin resistance to regulate the PI3K/Akt pathway and lipid metabolism, thereby resulting in reduced fat accumulation in the liver. The above results suggest that the intervention of *L. plantarum* ZDY2013 can hinder the progression of diet-induced NAFLD by reducing inflammation to regulate the PI3K/Akt pathway and regulating gut microbiota disturbance.

## 1. Introduction

Characterized by an overabundance of fat accumulation (with fat filtration being >5% of hepatocytes) and no evident alcohol consumption, non-alcoholic fatty liver disease (NAFLD) is a metabolic syndrome that ranges from simple hepar adiposum to non-alcoholic steatohepatitis and cirrhosis, and even to hepatocellular carcinoma [1]. A large number of clinical studies show that NAFLD is associated with heightened rates of both intrahepatic illness morbidity and mortality, as well as an augmented likelihood of various extrahepatic disorders [2]. Based on the statistics of global epidemiological research, the morbidity of NAFLD is about 22.1–28.65% of the world’s population, and the mortality rate of patients with NAFLD will increase progressively with worsening NAFLD; even simple fatty liver disease will increase the risk of death by 71% [3,4]. With the rapid increase of its prevalence, NAFLD poses a great threat to human health and has proved to be a worldwide public health issue [5]. However, in addition to lifestyle interventions such as diet interventions and regular exercise, there is a scarcity of drugs approved by regulatory agencies for the treatment of NAFLD [6]. Consequently, finding effective treatment is necessary to improve NAFLD.

At present, the widely accepted theory of the pathogenesis of NAFLD is a “multiple-hit model”, involving the interaction of genetic, neuroendocrine disorders, oxidative stress, inflammatory response, intestinal microbiota disorders, environmental factors, and alterations in the interorgan and intertissue communication dynamics [7]. Intestinal microbiota disorders, which are closely associated with inflammatory response, lipid deposition, oxidative stress, and insulin resistance (IR), play an indispensable role in the occurrence and development of NAFLD [8]. Intestinal microbiota disorders can promote intestinal inflammation to enlarge intestinal permeability, causing microbes, microbial products, and toxins to translocate from the gut into the hepar by means of the portal vein, thereby elevating the likelihood of NAFLD progression by increasing hepatic inflammation [9]. In addition, after the introduction of intestinal microbiota from obese mice, the energy absorption efficiency of sterile mice was improved, and the body weight was significantly increased, suggesting that the gut microbiota of obese mice can absorb more energy and have an important effect on the occurrence of obesity [10]. Therefore, regulating gut microbiota dysbiosis may be an effective strategy for therapeutic targeting to ameliorate NAFLD.

Probiotics can arrive at the gut in a vigorous state, thereby generating a salutary impact on health [11]. Probiotics also can ameliorate gut microbiota dysbiosis and decrease the production of detrimental metabolites; therefore, probiotic supplements have application potential in the treatment of NAFLD [12]. A preclinical study has suggested that probiotic replenishers could colonize the intestinal tract, increase beneficial microbiota, and improve intestinal endotoxemia, thereby ameliorating the liver inflammatory response and delaying the progression of NAFLD [13]. *L. plantarum* strains K2 and K6 ameliorated NAFLD by improving liver function, lipogenesis-related genes, and oxidative stress [14]. Furthermore, the study has shown that *L. plantarum* NA136 ameliorated NAFLD by regulating intestinal microbiota and reducing inflammation [15].

*Lactiplantibacillus plantarum* ZDY2013 was isolated from Chinese traditional fermented acid beans, which exhibited antioxidant, anti-inflammatory, and good intestinal colonization abilities [16]. In addition, *L. plantarum* ZDY2013 can regulate the intestinal microbiota and improve intestinal permeability to alleviate inflammation [17]. In preparing the experiment, we found that *L. plantarum* ZDY2013 has a good cholesterol-lowering ability. Therefore, this research aimed to elucidate the impact of *L. plantarum* ZDY2013 intervention on the development of diet-induced NAFLD, as well as to delineate the potential molecular mechanisms implicated.

## 2. Materials and Methods

### 2.1. Strain

Under 37 °C and anaerobic (5.0% carbon dioxide, 10% hydrogen, 85% nitrogen) conditions, *L. plantarum* ZDY2013 was cultured in sterile MRS broth (Shanghai Fusheng Industrial Co., Ltd., Shanghai, China) in an anaerobic incubator (Gene Science, San Diego, CA, USA) for 24 h. The bacterial liquid was centrifuged (5000× *g*, 5 min), and the thalli were washed with a sterile 1× PBS buffer. *L. plantarum* ZDY2013 was preserved in the China Center for Type Culture Collection, with the preservation number CCTCC NO: M 2014170.

### 2.2. Cholesterol-Lowering Capability

In a sterile MRS–CHOL medium, bacterial cultures were inoculated at a concentration of 2% (*v*/*v*) and incubated under the anaerobic conditions mentioned above (37 °C, 24 h). This medium was an MRS medium supplemented with 0.1 mg/mL cholesterol. The determination of the cholesterol-lowering ability of the strain referred to the method of Azat et al. [18].

### 2.3. Animals and Intervention

Moreover, 24 five-week-old SPF male C57BL/6n mice (Beijing Vital River Laboratory Animal Technology Co., Ltd., Beijing, China) were acclimated for one week under standard conditions at the animal facility of Nanchang Royo Biotech, Co., Ltd., Nanchang, China, with a 12 h light/dark cycle, during which food and water were freely available. After the adaptation period ended, all mice were randomly divided into three groups (*n* = 8/group) using a random number table. Mice in the ND group (normal diet group) were provided with a normal diet (19.5% protein, 4.6% fat, 2% fiber, SPF (Beijing) Biotechnology Co., Ltd., Beijing, China) and water. Mice in the MD group (model group) and the LD group (*L. plantarum* ZDY2013 administration group) were given an HFFC diet (40% fat, 22% fructose, 2% cholesterol, Trophic Animal Feed High-Tech Co., Ltd., Nantong, China) and normal drinking water [19]. Meanwhile, the ND group and the MD group were orally gavaged with 0.1 mL PBS, and the LD group was orally gavaged with 0.1 mL 1 × 10^9^ CFU/mL *L. plantarum* ZDY2013. The body weight of mice was transcribed every four days. Mice were euthanized with ether after intervention for 12 weeks. Liver samples, cecum contents, serum, and colons were gathered and saved at −80 °C. We referred to the method of Chen et al. to calculate the liver weight index and Lee’s index [20].

### 2.4. Biochemical Analyses in the Serum and Liver

Furthermore, 0.1 g liver samples were homogenized in 0.9 mL sterile normal saline, and the homogenized liquid was centrifuged (4 °C, 5000× *g*, 10 min). The levels of blood glucose (Kit Number: F006-1-1), high-density lipoprotein cholesterol (HDL-C, Kit Number: A112-1-1), low-density lipoprotein cholesterol (LDL-C, Kit Number: A113-1-1), total cholesterol (TC, Kit Number: A111-1-1), triglycerides (TG, Kit Number: A110-1-1), aspartate aminotransferase (AST, Kit Number: C010-2-1), alanine aminotransferase (ALT, Kit Number: C009-2-1), superoxide dismutase (SOD, Kit Number: A001-3-2), catalase (CAT, Kit Number: A007-2-1), glutathione (GSH, Kit Number: A006-2-1), and malondialdehyde (MDA, Kit Number: A003-1-2) were measured using biochemical kits. The above-mentioned kits all came from Nanjing Jiancheng Bioengineering Institute, Nanjing, China.

### 2.5. Inflammatory Cytokines and IR

The levels of serum lipopolysaccharide (LPS, Kit Number: MM-0634M1), insulin (INS, Kit Number: MM-0579M1), IKKβ (Kit Number: MM-44844M1), IκB-α (Kit Number: MM-45167M1), and NF-κB (Kit Number: MM-44130M1were measured by using ELISA kit (Jiangsu Meimian Industrial Co., Ltd., Yancheng, China). The levels of Beclin1 (Kit Number: HB-P9S3110X), ATG5 (Kit Number: HB-P9S2003X), and LC3-II (Kit Number: HB-P9S1485X) in serum were measured by using an ELISA kit (Huabang BIO Co., Ltd., Shanghai, China). Insulin resistance in mice was assessed using the homeostasis model assessment for insulin resistance (HOMA-IR) and the insulin sensitivity index (ISI) [21].

### 2.6. Histological Staining

Frozen liver samples were embedded in an OCT compound and cut to 8 μm thickness with the intention of conducting Oil Red O staining, Hematoxylin and Eosin (H&E) staining, or Masson’s trichrome staining on 8 μm-thick polyformaldehyde-fixed liver or colon tissue. The images of the stained slices were obtained by an upright microscope (NIKON Eclipse ci, Nikon Precision Co., Ltd., Shanghai, China). These processes were completed at Wuhan Servicebio Technology Co., Ltd. (Wuhan, China). To prevent overestimating the impact of *L. plantarum* ZDY2013 due to non-blind outcome assessment, histological staining images were assessed by three study members who were blinded to the experimental grouping.

### 2.7. 16S rRNA Sequencing

Initially, DNA extraction was performed on the cecal contents, which was then followed by purification and subsequent quantification processes. Afterward, the high-throughput sequencing protocol and subsequent analysis process referred to our laboratory’s previous research [22]. The specific sequencing process was carried out by Biomarker Tech (Beijing, China).

### 2.8. Western Blot

Proteins were abstracted from liver tissue using a lysis solution (Beyotime, Shanghai, China) with 0.5 mM PMSF and phosphatase inhibitors. Protein concentrations were determined to calculate the loading amount. SDS-PAGE was performed on proteins using a 10% acrylamide gel. The specific operation process of protein immunoblotting also referred to a previous study [23]. Antibodies against t-Akt, p-Akt, SREBP-1c, and β-actin were from Beyotime Biotech, Shanghai, China. The secondary antibodies were from Proteintech Group, Inc., Wuhan, China.

### 2.9. Gene Expression Analysis

Through the utilization of the RNA extraction kit (GenStar, Beijing, China), high-quality RNA was successfully obtained from the liver and colon. Subsequently, the cDNA synthesis was carried out with the aid of a cDNA synthesis kit (Takara, Otsu, Japan). Relative mRNA expression of inflammation, tight junction proteins, fat synthesis, and PI3K-Akt pathway-related genes in the colon and liver was assessed by a three-step PCR reaction procedure and normalized using β-actin level as the standard [23]. Supplementary Table S1 lists the sequences of the primers.

### 2.10. Statistical Analysis

Data were analyzed by GraphPad Prism 8 software, provided by GraphPad Software, located in La Jolla, CA, USA. All results are exhibited as the means ± standard deviation (S.D.). One-way ANOVA was used to evaluate the differences between groups, and we subsequently performed Tukey’s HSD for multiple pairwise comparisons. *p* = 0.05 was the significance threshold. * *p* < 0.05; ** *p* < 0.01; *** *p* < 0.001; **** *p* < 0.0001.

## 3. Result

### 3.1. Cholesterol-Lowering Capability

The cholesterol removal rate of *L. plantarum* ZDY2013 is 63.3%, which is superior to that of *L. rhamnosus* GG (Table 1). The survival rates of *L. plantarum* ZDY2013 and LGG in MRSC broth were 79.6% and 71.7%, respectively. The above results indicated that *L. plantarum* ZDY2013 might have a good cholesterol-lowering capability.

### 3.2. L. plantarum ZDY2013 Intervention Reduced the Liver Fat Accumulation in Mice Fed with HFFC Diet

**Figure 1:**
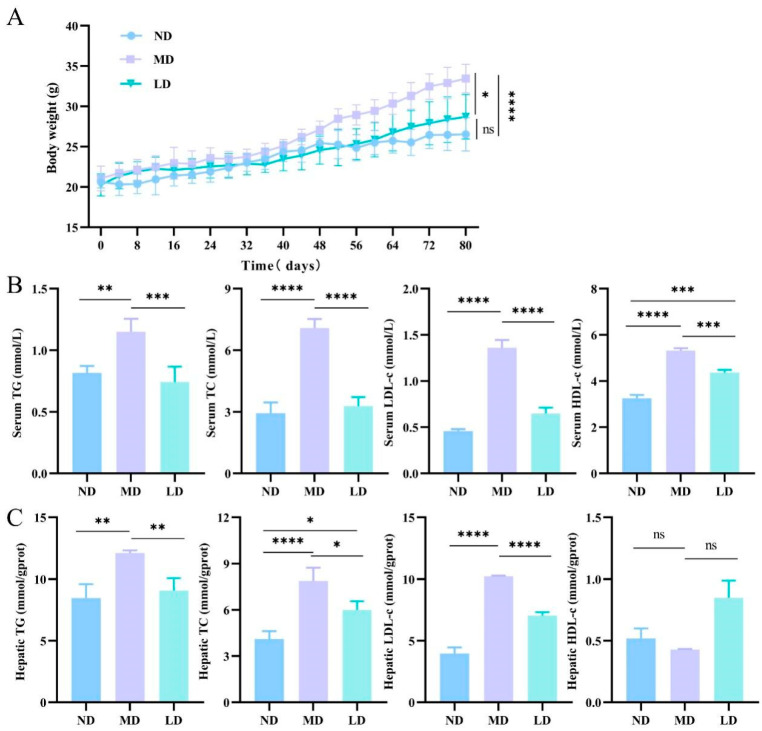
Impacts of *L. plantarum* ZDY2013 on body weight and biochemical parameters (**A**) Changes in body weight of mice; the levels of TC, TG, HDL-C and LDL-C in serum (**B**) and liver (**C**). * *p* < 0.05; ** *p* < 0.01; *** *p* < 0.001; **** *p* < 0.0001; ns, not significant.

Hepatic lipid deposits were assessed to determine whether the NAFLD model was successful. In the MD mice, the liver weight, Lee’s coefficient, and liver index were evidently greater than in the ND mice. However, in the LD mice, the intervention of *L. plantarum* ZDY2013 led to observably lower levels of the above indicators compared to the MD mice (Table 2). The weight gain of mice was evidently higher in the MD group than the ND group, but that in the LD group was obviously lower in comparison with the MD group (Figure 1A). The blood lipid levels in the MD mice were evidently higher than in the ND mice, while the LD mice exhibited observably lower levels of TC, TG, and LDL-C than the MD mice (Figure 1B,C). Moreover, the differences in the HDL-C levels of the liver among all groups were not significant. The above findings exhibit that the long-term intake of an HFFC diet caused an obvious increase in body weight and liver lipids in mice.

### 3.3. L. plantarum ZDY2013 Intervention Alleviated Liver Dysfunction in Mice Fed with HFFC Diet

**Figure 2:**
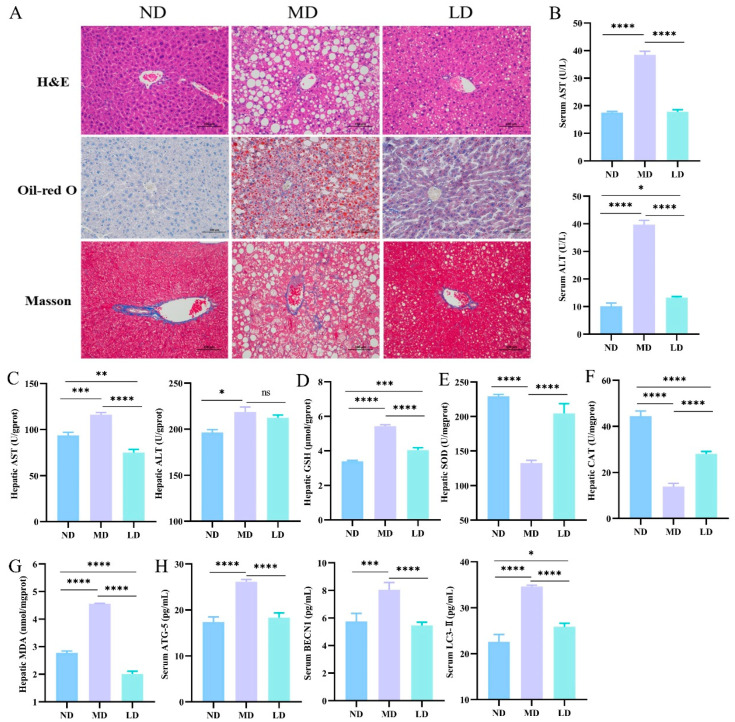
Impacts of L. plantarum ZDY2013 on hepatic steatosis; liver function and oxidative stress (**A**) hepatic H&E staining, Oil Red O staining and Masson staining (200×); (**B**) serum and (**C**) hepatic AST and ALT levels; the activities of hepatic (**D**) GSH, (**E**) SOD, (**F**) CAT and (**G**) MDA; (**H**) the concentration of autophagic pathway-related proteins in serum. * *p* < 0.05; ** *p* < 0.01; *** *p* < 0.001; **** *p* < 0.0001; ns, not significant.

In this study, H&E staining, Oil Red O staining, and Masson staining of liver samples were used to assay histological changes. As shown in Figure 2A, moderate-to-marked macrovesicular steatosis was developed in the MD mice via H&E staining, whereas the steatosis grade was great lower in the LD group. Compared with the ND mice, the MD mice showed a notable accumulation of liver lipid droplets, as revealed by Oil Red O staining, while the LD mice were significantly lower in liver lipid droplets compared to the MD mice. Masson staining revealed that, compared to the ND mice, the MD mice exhibited significant liver collagen deposition, while the LD mice showed significantly lower liver collagen deposition compared to the MD mice.

By measuring the enzymatic activities (AST and ALT), we investigated whether *L. plantarum* ZDY2013 could alleviate liver dysfunction in mice fed with an HFFC diet. Compared with the ND mice, the MD mice had significantly lower activities of AST and ALT, while those were similar in the LD and ND groups.

### 3.4. L. plantarum ZDY2013 Intervention Relieved Hepatic Oxidative Stress and Inhibited Autophagic Pathway in Mice Fed with HFFC Diet

The reduction in antioxidant capacity could result in lipid peroxidation and hepatic damage [24]. To explore the impact of *L. plantarum* ZDY2013 on liver oxidative stress, the activities of SOD and CAT, as well as the levels of GSH and malondialdehyde MDA, in the liver were assessed. In contrast to the ND group, mice exhibited lower SOD and CAT activities along with higher GSH and MDA content in the MD group (Figure 2D–G). However, the related conditions were alleviated in the LD group mice.

In MD mice, the content of autophagy proteins was evidently higher compared with the ND group, including Beclin1, ATG5, and LC3-II. Nevertheless, the content of these proteins in the LD group with *L. plantarum* ZDY2013 administration was observably lower compared with the MD group (Figure 2H). The above results present that *L. plantarum* ZDY2013 could relieve autophagy disorders in mice fed with an HFFC diet.

### 3.5. L. plantarum ZDY2013 Intervention Improved IR in Mice Fed with HFFC Diet

**Figure 3:**
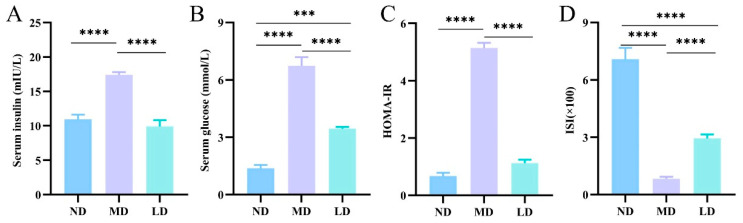
Impacts of *L. plantarum* ZDY2013 on insulin resistance (**A**) Serum insulin; (**B**) blood glucose; (**C**) HOMA-IR; (**D**) ISI. *** *p* < 0.001; **** *p* < 0.0001.

Insulin resistance is a hallmark of NAFLD, which is important to the pathogenesis and progression of diseases associated with obesity. Moreover, IR is an important factor in determining the progression from NAFLD to NASH [25]. In elucidating the impact of *L. plantarum* ZDY2013 administration on IR in mice fed with an HFFC diet, we detected the content of serum glucose and insulin. Compared to the ND mice, the MD mice exhibited significantly higher levels of blood glucose, insulin content, and HOMA-IR but lower ISI values. Compared with the MD mice, the LD mice showed evidently lower levels of blood glucose, insulin content, and HOMA-IR, while ISI was higher (Figure 3A–D). Therefore, *L. plantarum* ZDY2013 improved IR in mice fed with an HFFC diet.

### 3.6. L. plantarum ZDY2013 Intervention Regulated Intestinal Microbiota Disturbance in Mice Fed with HFFC Diet

**Figure 4:**
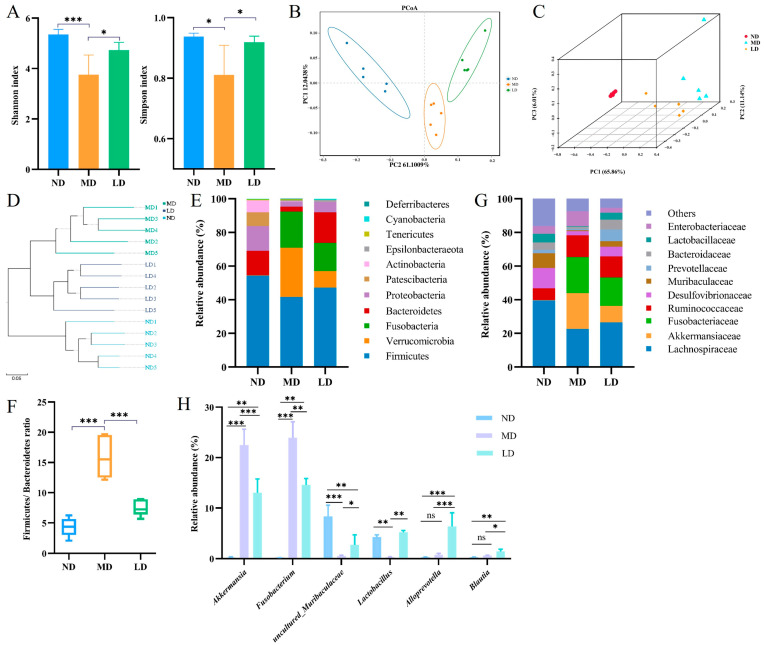
Impacts of *L. plantarum* ZDY2013 on intestinal microbiota disturbance (**A**) Shannon and Simpson index; (**B**) PCoA plot; (**C**) Three-dimensional PCoA analysis chart; (**D**) UPGMA hierarchical clustering analysis; the intestinal bacterial composition at the levels of (**E**) the phylum, (**G**) family and (**H**) genus; (**F**) *Firmicutes*/*Bacteroidetes* ratio. * *p* < 0.05; ** *p* < 0.01; *** *p* < 0.001; ns, not significant.

Alpha and beta diversity were analyzed to assess the intestinal microbiota differences among the groups. The MD group had significantly lower alpha diversity indexes (Shannon, 3.75 ± 0.70, and Simpson, 0.81 ± 0.09) than the ND group (Shannon, 5.35 ± 0.18, and Simpson, 0.93 ± 0.01). After the intervention of *L. plantarum* ZDY2013, the two alpha diversity indices were evidently higher in comparison with the MD group (Figure 4A). β-diversity analysis (PCoA and UPGMA) showed that the composition of intestinal microbiota in the LD group mice was analogous to the ND group mice but inconsistent with the MD group mice (Figure 4B–D).

Through cluster analysis, the relative abundance of different taxa among different samples was detected. Dominant phyla in these samples included *Firmicutes*, *Bacteroidetes*, *Proteobacteria*, and *Fusobacteria*. In contrast to the ND mice, the abundances of *Firmicutes*, *Bacteroidetes*, and *Proteobacteria* were lower, while the abundances of *Verrucomicrobia* and *Fusobacteria* were higher in the MD mice. In contrast to the MD mice, the LD mice revealed higher abundances of *Firmicutes*, *Bacteroidetes*, and *Proteobacteria*, while the abundance of *Fusobacteria* and *Verrucomicrobia* was lower (Figure 4E). Compared to the LD mice, the lower abundance of *Bacteroidetes* resulted in a higher *Firmicutes*/*Bacteroidetes* (F/B) ratio in the MD mice (Figure 4F). In contrast to the ND mice, *Lachnospiraceae*, *Desulfovibrionaceae*, *Prevotellaceae*, and *Muribaculaceae* showed lower abundance, whereas *Akkermansiaceae* and *Fusobacteriaceae* showed higher abundance at the family level in the MD mice. *L. plantarum* ZDY2013 administration recovered the microbiota structure at the family level (Figure 4G). Compared with the ND mice, at the genus level, the abundances of *Fusobacterium*, *Ruminococcaceae*_UCG-005, and *Akkermansiaceae* were significantly higher, whereas the abundances of *Lactobacillus*, *Blautia,* and *uncultured*_*bacterium*_*f*_*Muribaculaceae* were evidently lower in the MD mice. By contrast, *L. plantarum* ZDY2013 regulated the microbiota structure, particularly increasing the abundance of *Alloprevotella* (Figure 4H).

### 3.7. L. plantarum ZDY2013 Intervention Relieved Intestinal Inflammation in Mice Fed with HFFC Diet

**Figure 5:**
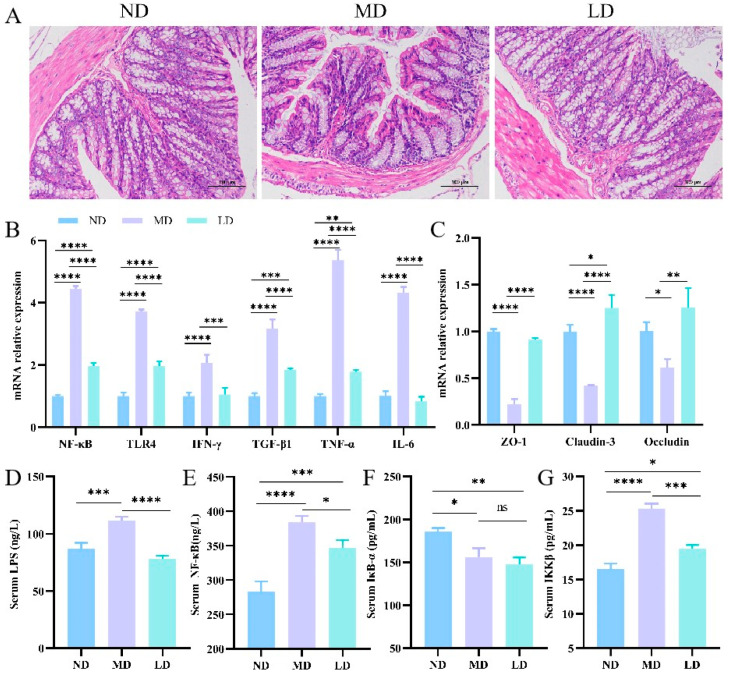
Impacts of *L. plantarum* ZDY2013 on the intestinal inflammation (**A**) colonic H&E staining; (**B**) mRNA levels of NF-κB inflammation pathway cytokines and (**C**) the tight junctional protein in the colon; the concentration of (**D**) LPS, (**E**) NF-κB, (**F**) IκB-α, and (**G**) IKKβ in serum. * *p* < 0.05; ** *p* < 0.01; *** *p* < 0.001; **** *p* < 0.0001; ns, not significant.

In general, intestinal microbiota disorders are accompanied by inflammation and intestinal barrier destruction. The extent of pathological damage in colonic tissue was evaluated utilizing H&E staining. The MD group revealed a pronounced destruction of the colon mucosa, accompanied by a significant infiltration of inflammatory cells, in contrast to the ND group (Figure 5A). In addition, *L. plantarum* ZDY2013 intervention showed less severe mucosal damage and slighter inflammatory cell infiltration compared with the MD group. Therefore, in the mice fed with an HFFC diet, *L. plantarum* ZDY2013 intervention could relieve colon pathological damage.

The mRNA quantitation of colonic inflammatory factors revealed that, in the MD mice, the expression of NF-κB, TLR4, IκB-α, IFN-γ, TNF-α, and IL-6 was notably higher compared with the ND mice. The aforementioned expressions in the LD mice were drastically lower than those in the MD mice (Figure 5B). To explore whether mice with NAFLD had intestinal barrier destruction and research the impact of *L. plantarum* ZDY2013 on the permeability of the gut, the mRNA expression of colonic TJ proteins was detected. The mRNA expression levels of Claudin-3, Occludin, and ZO-1 were lower in the MD mice in comparison with the ND mice, while such levels in the LD mice were higher than in the MD mice (Figure 5C).

Intestinal barrier trauma may cause the seepage of LPS and pro-inflammatory molecules out of the gut [26]. The serum LPS level was remarkably higher in the MD mice than the ND mice due to the HFFC diet. However, it was distinctly lower after the intervention with *L. plantarum* ZDY2013 compared to the MD mice (Figure 5D). In contrast to the ND group, the MD group exhibited significantly lower serum IκB-α activity but higher activities of NF-κB and IKKβ. Nevertheless, the activities of NF-κB and IKKβ were lower than in the MD mice due to *L. plantarum* ZDY2013 intervention in the LD mice. Moreover, in the LD and MD groups, the serum IκB-α levels were similar (Figure 5E–G). The above findings indicate that *L. plantarum* ZDY2013 intervention promoted the mRNA expression of TJ proteins to prevent LPS and inflammatory cytokine leakage from the gut.

### 3.8. L. plantarum ZDY2013 Intervention Relieved Liver Inflammation and Regulated Lipogenesis through Regulating the PI3K/Akt Signaling Pathway

**Figure 6:**
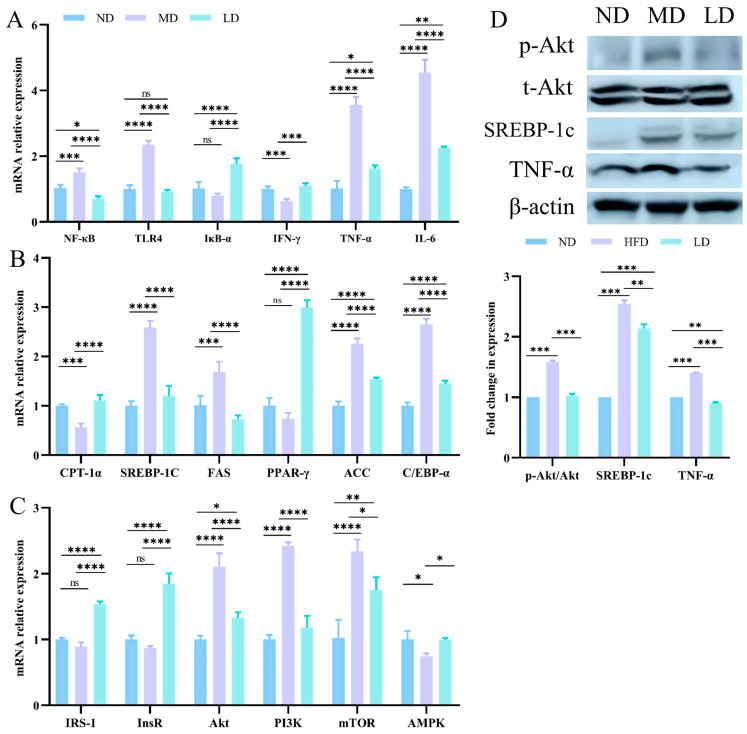
Impacts of *L. plantarum* ZDY2013 on hepatic inflammatory responses and lipid metabolism (**A**) mRNA levels of inflammation-related genes; (**B**) mRNA levels of lipogenesis and lipid oxidation-related genes; (**C**) mRNA levels of PI3K-Akt pathway-related genes in the liver; (**D**) Western blot. * *p* < 0.05; ** *p* < 0.01; *** *p* < 0.001; **** *p* < 0.0001; ns, not significant.

To explore whether *L. plantarum* ZDY2013 administration could attenuate liver inflammation in mice fed with an HFFC diet, the expression of hepatic inflammation-related mRNA was detected. In contrast to the ND mice, the mRNA expression of NF-κB, TLR4, IκB-α, IFN-γ, TNF-α, and IL-6 was evidently higher in the MD mice. However, with *L. plantarum* ZDY2013 intervention, the LD mice exhibited significantly lower expression levels of the above factors compared to the MD mice (Figure 6A).

Hepatic lipid metabolism is primarily regulated by multiple associated pathways, such as fatty acid synthesis pathways and β-oxidation [27]. β-oxidation can indicate hepatic lipid accumulation, as it is the critical way in lipid metabolism [28]. The mRNA expression of genes concerning lipid oxidation (PPAR-γ and CPT-1α), lipogenesis (FAS, ACC, C/EBP-α, and SREBP-1c), and PI3K/Akt pathway-related genes were investigated to explore the impact of *L. plantarum* ZDY2013 on lipid deposition in the liver. In contrast to the ND mice, the mRNA expression level of CPT1α was significantly lower in the MD mice, while the mRNA expression of ACC, C/EBP-α, FAS, and SREBP-1c was significantly higher. However, in contrast to the MD mice, the mRNA expression of CPT1α and PPAR-γ in the LD mice was significantly higher, while the mRNA expression of ACC, C/EBP-α, FAS, and SREBP-1c was significantly lower (Figure 6B). Similarly, *Lactobacillus plantarum* LG42 can effectively downregulate the expression of the ACC gene in adipose tissue to inhibit fatty acid synthesis [29]. These findings implied that the capability of *L. plantarum* ZDY2013 to decrease lipid accumulation might be related to the downregulation of lipogenesis. Compared to the ND mice, in the MD mice, the mRNA expression of AMPK was lower, whereas that of Akt, PI3K, and mTOR was higher. Following the intervention with *L. plantarum* ZDY2013, the mRNA expression of IRS-1, InsR, and AMPK was higher compared to the MD mice, while the mRNA expression of Akt, PI3K, and mTOR was lower (Figure 6C). The expression levels of TNF-α, t-Akt, p-Akt, SREBP-1c, and β-actin proteins in the liver tissue were detected by Western blotting. The expression of TNF-α, p-Akt, and SREBP-1c proteins was higher than the ND mice in the MD mice, and it was lower than the MD mice in the LD mice (Figure 6D).

## 4. Discussion

NAFLD represents a prevalent chronic liver condition affecting individuals across various age demographics, and the proportion of adult NAFLD accounts for 30% [30]. With the pivotal function of the gut-liver axis in NAFLD’s onset and development becoming clearer, investigations about NAFLD prevention and therapy using probiotics have been increasing continuously [31]. In this study, *L. plantarum* ZDY2013 intervention inhibited the development of HFFC diet-induced NAFLD through regulating gut microbiota and LPS/NF-κB and PI3K/Akt pathways to reduce fat accumulation in the liver.

At present, many researchers indicate that the intestinal microbiota exerts an essential function in the development of NAFLD, involving enhancing mucosal immunity, improving intestinal barrier integrity, and reducing the leakage of harmful metabolism [32]. Significantly, the high proportion of F/B indicates that the host can absorb more energy from the food and promote fat accumulation [33]. In this research, compared to the mice in the ND mice, the mice in the MD mice that consumed the long-term HFFC diet exhibited lower gut microbial diversity, a higher F/B ratio, and a higher abundance of *Enterobacteriaceae* and *Fusobacterium*, and the results are consistent with most studies [34]. *Enterobacteriaceae* produces LPS and promotes proinflammatory cytokine production, which is associated with the exacerbation of hepatic inflammation [35]. Furthermore, *Fusobacterium* can damage the intestinal barrier and result in high expression levels of LPS and TNF-α in patients with NAFLD, which may induce insulin resistance [36]. However, *L. plantarum* ZDY2013 intervention led to a lower F/B ratio and a lower abundance of *Enterobacteriaceae* and a higher abundance of beneficial gut microbiota—for instance, *Lachnospiraceae*, *Muribaculaceae*, *Lactobacillus*, *Alloprevotella*, and *Blautia*. It is well known that *Lactobacillus*, *Blautia*, and *Alloprevotella* can ameliorate intestinal barrier integrity and the leakage of LPS, and competitively inhibit pathogenic bacterial growth [37,38,39]. Furthermore, *Lachnospiraceae* and *Muribaculaceae* synthesize short-chain fatty acids (SCFAs) to maintain intestinal permeability, exhibiting potential anti-inflammatory activity that can enhance insulin sensitivity and improve IR [40,41]. Multiple researchers have demonstrated that providing SCFAs mitigates diet-induced NAFLD by decreasing inflammation and improves intestinal barrier function and hepatic lipid metabolism [42,43]. Therefore, *L. plantarum* ZDY2013 intervention might restrain the development of NAFLD by increasing the beneficial intestinal bacteria and regulating intestinal microbiota dysbiosis induced by the consumption of an HFFC diet.

Gut microbiota dysbiosis could cause many problems, including the overgrowth of pathogenic or harmful bacterial and intestinal barrier damage in patients with NAFLD [44]. Intestinal barrier injury, characterized by the breakdown of the intestinal epithelium and the upward serum LPS level, is linked to the progression of NAFLD [45]. Here, compared with the ND group, a lower mRNA expression of ZO-1, Claudin-3, and Occludin was observed in the MD group. Compared with the ND group, the remarkably higher serum LPS level reflected an elevation of intestinal permeability in the MD group. The results of intestinal microbiota analysis indicated that intestinal microbiota dysbiosis induced by the consumption of an HFFC diet could ultimately weaken the intestinal barrier. Once the intestinal barrier is disrupted, LPS and inflammation-related factors produced by microbiota will reach the liver through blood circulation, promoting liver inflammation, oxidative stress, IR, and the disturbance of lipid metabolism [46]. LPS binding to TLR4 will activate NF-κB to increase inflammatory cytokines, thereby resulting in hepatic injury and inflammation [47]. As shown in our results, the liver inflammation in the MD mice was significantly higher than in the ND mice. However, in contrast to the MD mice, *L. plantarum* ZDY2013 intervention could maintain liver inflammation at a lower level by repairing the barrier of the gut, thwarting the leakage of LPS, and inhibiting the NF-κB pathway in the LD mice.

TNF-α and IL-6 interfere with insulin signals at the level of insulin receptors through multiple signal pathways, leading to IR [48]. In previous reports, the interplay of insulin with its receptor triggered the activation of IRS-1, PI3K, and Akt [49]. The PI3K-Akt pathway is vital for glycolipid metabolism because it is the primary pathway of insulin signaling [50]. However, the PI3K-Akt pathway will be over-activated in the inflammatory state of NAFLD mice [51]. Subsequently, the activation of Akt signaling activates SREBP-1c and its downstream lipases (ACC and FAS) to increase adipogenesis, aggravating liver tissue damage [52]. In the MD mice, Akt was activated, and lipid synthesis and IR were greater in contrast to the ND mice; contrarily, those improved in the LD mice, thereby decreasing lipid synthesis. Therefore, in the mice fed with an HFFC diet, *L. plantarum* ZDY2013 might inhibit the PI3K-Akt pathway to relieve IR and decrease lipid synthesis. This may be related to the fact that *L. plantarum* ZDY2013 can regulate the gut microbiota to reduce inflammation in hepatic tissues.

Investigations have disclosed that diets high in fat and fructose elevate levels of free radicals and reactive oxygen species, leading to oxidative stress—a critical point in the advancement of NAFLD [53]. Furthermore, previous work has shown that oxidative stress exacerbates hepatic de novo lipogenesis, causing the vicious cycle of steatosis and damage [54]. MDA is regarded as an indicator of oxidative stress, which is produced by lipid peroxidation. Moreover, SOD is capable of transforming oxygen free radicals into hydrogen dioxide and oxygen [55]. In this research, the LD mice showed higher antioxidant activity than the MD mice. The MDA level was lower in the LD mice than in the MD mice after the intervention with *L. plantarum* ZDY2013, and these results also appeared in another study using *L. plantarum* NA 136 [15]. Autophagy is a pivotal process that maintains liver physiology and balances liver metabolism [56]. Within the liver, autophagy is able to stabilize the protein and lipid levels; however, it may also lead to injury [57]. In this study, HFFC diet consumption could induce autophagy disorders, whereas *L. plantarum* ZDY2013 intervention alleviated autophagy maladjustment in the mice fed with an HFFC diet, and the results are consistent with previous studies [58]. Based on previous studies, oxidative stress and inflammation can lead to liver injury [59]. The measurement of liver ALT and AST, whose continuously elevated serum levels can indicate NAFLD, can allow us to detect hepatocellular damage [60]. Our results showed that long-term HFFC diet consumption increased oxidative stress and caused autophagy maladjustment in the NAFLD mouse model, which resulted in liver damage in NAFLD-model mice, whereas the administration of *L. plantarum* ZDY2013 could alleviate liver damage in mice.

In this research, we discovered the inhibitory impact of *L. plantarum* ZDY2013 on the development of NAFLD by administering it to mice fed with an HFFC diet and elucidated the underlying molecular mechanisms. However, the impact of *L. plantarum* ZDY2013 on mice that are already diagnosed with NAFLD was not explored, which warrants further investigation.

## 5. Conclusions

In summary, the results suggest that regulating the intestinal microbiota and restoring the barrier of the gut to inhibit the LPS/NF-κB signaling pathway may be the potential underlying mechanism for *L. plantarum* ZDY2013 restraining the progression of NAFLD, thereby decreasing inflammatory cytokine levels, relieving IR, regulating the PI3K/Akt pathway to decrease hepatic lipid accumulation, restoring liver function, and ameliorating oxidative stress. The aforementioned results suggested that *L. plantarum* ZDY2013 could be used as a potential intervention to inhibit the development of NAFLD.

## Figures and Tables

**Table 1:** The cholesterol-lowing capability of *L. plantarum* ZDY2013.

Items	*L. plantarum* ZDY2013	LGG
The cholesterol-clearance rate (%)	63.3 ± 0.01 *	57.7 ± 0.04
The survival rate (%)	79.6 ± 0.27 *	71.7 ± 0.26

* *p* < 0.05; * is compared with LGG.

**Table 2:** Liver weight and Lee’s index.

Items	ND	MD	LD
Liver weight (g/each)	1.18 ± 0.05 ***	1.54 ± 0.13	1.24 ± 0.09 ***
Liver index (%)	4.44 ± 0.27 *	4.80 ± 0.16	4.27 ± 0.15 **
Lee’s index	3.08 ± 0.05 ***	3.39 ± 0.06	3.25 ± 0.06 **

* *p* < 0.05; ** *p* < 0.01; *** *p* < 0.001; * is compared with MD.

## Data Availability

The datasets generated during the study are available in the National Center for Biotechnology Information (NCBI) under accession number PRJNA856471.

## Supplementary Materials

The following supporting information can be downloaded at: https://www.mdpi.com/article/10.3390/nu16070958/s1, Table S1: Primer sequences for quantitative Real-Time polymerase chain reaction.
