# Supplementary material for: Lactiplantibacillus plantarum ZDY2013 Inhibits the Development of Non-Alcoholic Fatty Liver Disease by Regulating the Intestinal Microbiota and Modulating the PI3K/Akt Pathway
Source: Nutrients. 2024 Mar 27;16(7):958. doi: 10.3390/nu16070958 (PMC11013082; doi:10.3390/nu16070958)
Supplement: Supplementary file 1 [file nutrients-16-00958-s001.zip › nutrients-2873183-supplementary.pdf]

Supplementary Table S1 Primer sequences for quantitative real-time polymerase chain reaction.

Gene		Sequence
β-actin	Forward	GCTCCTCCTGAGCGCAAGTA
	Reverse	CAGCTCAGTAACAGTCCGCC
IκB-α	Forward	GAAGAGAAGCCGCTGACCAT
	Reverse	CAGAAGTGCCTCAGCAATTCC
NF-κB	Forward	ACGATCTGTTTCCCCTCATC
	Reverse	TGCTTCTCTCCCCAGGAATA
IFN-γ	Forward	TGATTGCGGGGTTGTATCTG
	Reverse	CTGTCTGGCCTGCTGTTAAA
TNF-α	Forward	CCCTCACACTCAGATCATCTTCT
	Reverse	GCTACGACGTGGGCTACAG
IL-6	Forward	CTGCAAGAGACTTCCATCCAG
	Reverse	AGTGGTATAGACAGGTCTGTTGG
TGF-β1	Forward	GTCACTGGAGTTGTACGGCA
	Reverse	TCATGTCATGGATGGTGCCC
TLR4	Forward	AGCAAAGTCCCTGATGACATT
	Reverse	CAGCCACCAGATTCTCTAAAC
FAS	Forward	ATCCAACTATGGCTTCGC
	Reverse	GCTGTTCGCAAATACGCT
SREBP-1C	Forward	GCGGAGCCATGGATTGCAC
	Reverse	CTCTTCCTTGATACCAGGCCC
ACC	Forward	CAATCCTCGGCACATGGAGA
	Reverse	GCTCAGCCAAGCGGATGTAGA
CPT-1α	Forward	TGAGCGACTGGTGGGAGGAG
	Reverse	GAGCCAGACCTTGAAGTAGCG
C/EBPα	Forward	AGCAACGAGTACCGGGTACG
	Reverse	TGTTTGGCTTTATCTCGGCTC
PPAR-γ	Forward	GATGCACTGCTATGAGCACTT
	Reverse	AGAGGTCCACAGAGCTGATCC
IRS-1	Forward	GTTGAGTTGGGCAGAATAGG
	Reverse	CAGCAAGGAAGAGTGAGTAG
InsR	Forward	CGCTCCTATGCTCTGGTAT
	Reverse	GAGTGATGGTGAGGTTGTGT

Gene		Sequence
PI3K	Forward	GAAACAAAGCGGAGAACCTAT
	Reverse	CTTGACTTCGCCGTCTACCAC
Akt	Forward	ACAGTCATTGAGCGCACCT
	Reverse	CCTGATCGGAAGTCCATCGTCT
mTOR	Forward	TCGGTGCAAACCTACAGAAGC
	Reverse	TGCAGGTCGTATATGGACAGAG
AMPK	Forward	GTTGTAAACCCCTATTATTTGCGTG
	Reverse	TGGAGTAGCAGTCCCTGATTTGG
ZO-1	Forward	GATCCCTGTAAGTCACCCAGA
	Reverse	CTCCCTGCTTGCACTCCTATC
Occludin	Forward	GGACTGTCAACTCTTTCCGC
	Reverse	CATTTATGATGAACAGCCCC
Claudin-3	Forward	CTGTCTGTCCTCTTCCAGCC
	Reverse	CCACTACCAGCAGTCGATGA
